# Membrane-Embedded Anti-Cancer Peptide Causes a Minimal Structural Perturbation That Is Sufficient to Enhance Phospholipid Flip-Flop and Charge Permeation Rates

**DOI:** 10.3390/life15071007

**Published:** 2025-06-25

**Authors:** Alfredo E. Cardenas, Ron Elber

**Affiliations:** The Oden Institute, University of Texas at Austin, Austin, TX 78712, USA; alfredo@ices.utexa.edu

**Keywords:** membrane permeation, cell penetrating peptide, membrane defect

## Abstract

A prime role of biological membranes is to form barriers for material transport into and out of cells. Membranes consist of phospholipids with polar heads, which are presented to the aqueous solutions, and hydrophobic tails that form the membrane core. This construct prevents the permeation of hydrophilic, well-solvated molecules across the lipid hydrophobic barrier. The barrier is not absolute, and several approaches are available for efficient translocation. Channels and pumps enable selective and efficient transport across membranes. Another transport mechanism is passive permeation, in which permeants, without assistance, directly transport across membranes. Passive transport is coupled to transient defects in the membrane structure that make crossing the hydrophobic bilayer easier—for example, displacements of head groups from aqueous solution–membrane interface into the membrane core. The defects, in turn, are rare unless assisted by passively permeating molecules such as cell-penetrating peptides that distort the membrane structure. One possible defect is a phospholipid molecule with a head pointing to the hydrophobic core. This membrane distortion allows head group flipping from one layer to the other. We show computationally, using atomically detailed simulations and the Milestoning theory, that the presence of a cell-penetrating peptide in a membrane greatly increases phospholipid flip-flop rate and hence defect formation and the permeability of membranes.

## 1. Introduction

**Figure 1:**
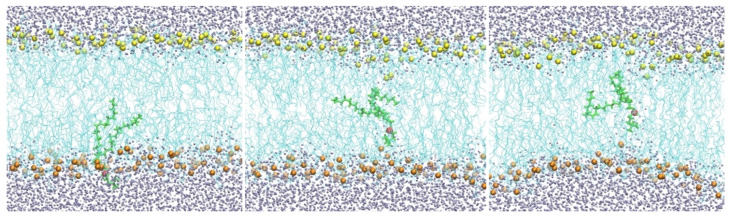
A membrane with a hydrophobic core of DOPC lipids (cyan lines). Oxygen atoms of water molecules are shown as gray spheres, and the phosphate groups as yellow spheres (top) and orange spheres (bottom). Also shown is a single phospholipid molecule (green) with the phosphate head (red) migrating from the water–membrane interface to the membrane center from left to right.

Biological membranes are highly heterogeneous systems [1,2]. They are primarily made of phospholipids, but many inserts, such as transmembrane proteins, are also present. Each of the phospholipid molecules is bi-functional. The phosphate head is charged or polar, while the lipid tail consists of hydrocarbon chain(s) and is hydrophobic. Phospholipid molecules form bilayers in which the polar head groups point to the aqueous solutions. A membrane with a hydrophobic core (cyan lines) of DOPC lipids is shown in (Figure 1).

The lipids form a thin apolar layer of ~4 nm width and pose a barrier for translocation across the membrane. This enables living systems to monitor material transport in and out of the cell and use transport machinery such as channels and pumps embedded in the membrane to control passage.

Membranes exist as two-dimensional fluids in which the phospholipid molecules diffuse rapidly in the membrane layer on the nanosecond time scale but rarely cross between the layers, a process called flip-flop [3,4]. The two layers of biological membranes have asymmetric composition. Why is it challenging to flip between the layers? The difficulty in conducting a flip-flop motion is that the polar heads must cross an apolar environment between two aqueous solutions, which is electrostatically penalized. The cost is therefore similar to the translocation of a charge across a membrane. How to overcome this barrier? One approach uses biological machinery such as flippases, floppases, and scramblases [5] that translocate phospholipids from one layer to the other. This process requires biological energy.

We do not know the detailed molecular mechanisms of the translocating enzymes. However, from a mechanical viewpoint, the translocation between the layers would have been easier if the hydrophobic layer had been disrupted, creating a polar defect that can assist the migration of the heads. For example, a phospholipid head is displaced to the lipid core. Such a defect can form spontaneously as a thermal fluctuation, which will be exceptionally rare and slow. Nevertheless, given a defect that places polar groups along the diffusion pathway between the outer and inner leaflet, flip-flop motions will be easier and faster.

What kind of membrane defects accelerate flip-flops? Large membrane breaks are likely to do so. Indeed, proposals for membrane pores and channels that assist flip-flop motions have been published [6,7]. It is not surprising that large, extensive damage to the membrane integrity exposes more phospholipid molecules to the aqueous solution and enhances their flip-flop rate. One could imagine a diverse set of membrane perturbations that address the goal. Perhaps a more focused question would be, what is the minimal membrane defect that can significantly enhance the flip-flop rate? In a recent paper, it was demonstrated that a peptide on a membrane surface accelerates a flip-flop rate [8].

In several publications, we have shown that cell-penetrating peptides (CPP) that interact with the surface of the membrane, or permeate it, cause local defects. In contrast to transmembrane proteins that fit well geometrically in phospholipid membranes, the structural complementarity of CPP with the membrane is imperfect. The defects mix phosphate head-groups with lipid hydrocarbon chains [9,10] at the membrane core, and support the migration of charged peptides across the membrane. We have introduced the DAC model of peptide–membrane interactions (Defect Assisted by Charge [10]), which explains permeation efficiency. The defect is an essential component of the permeation mechanism. During the permeation, the polar phospholipid heads follow the peptide’s positively charged residues into the membrane center [10]. Here, we ask a reverse question. Consider a defect induced by a single permeating peptide. Is it sufficient to reduce the free energy barrier of phospholipid flip-flop? In this paper, we illustrate that the effect is dramatic.

The perturbation induced by a single peptide is clearly smaller than the distortion caused by an aggregate of several peptides, or a carpet of peptides on the membrane surface [11]. To model the impact of an individual molecule, we consider the anti-cancer peptide NAF-1^44–67^, embedded in a DOPC membrane, a system with which we have considerable prior experience [12]. NAF-1 is the name of the protein from which the peptide is derived, and 44–67 is the sequence of the peptide in the protein. Our previous investigations suggest that the peptide center of mass is at a broad minimum in the membrane core. The present investigation, therefore, asks, given that the peptide is at the membrane center (we restrain it there), what is the rate of phospholipid flips?

## 2. Methods

The membrane system and initial input files were prepared with the Membrane Builder option of CHARMM-GUI [13]. The two bilayer membranes contain 160 DOPC molecules. One of the membrane patches was built with an NAF-1^44–67^ molecule inserted at the membrane center, while the second model had only DOPC molecules. The initial configuration of NAF-1^44–67^ was taken from an earlier work [12]. A total of 10,743 water molecules were included in the simulation box to fully hydrate the membranes. The dimensions of the simulation boxes were 7.4 × 7.4 × 7.9 nm^3^ and 7.7 × 7.7 × 9.0 nm^3^ for the pure DOPC membrane system and the membrane with an NAF-1^44–67^ molecule, respectively. Potassium and chloride ions were added for an ionic strength of 0.15 M.

The system with NAF-1^44–67^ at the membrane center has about one Cl^−1^ at the membrane core during the simulations detailed in Results and Discussions. No Cl^−1^ ions were detected at the core of the pure DOPC system. A small number of K^+^ ions permeates both membrane systems. The simulations were conducted with GROMACS version 2021.4 [14]. The CHARMM36m force field was used for the lipid molecules and NAF-1^44–67^ [15]. Water molecules were modeled with CHARMM TIP3P [16]. Periodic boundary conditions were applied to all three spatial directions. The particle mesh Ewald method [17] computed the long-range electrostatic interactions with a real space cutoff of 1.2 nm, while the van der Waals interactions were modelled with Lennard-Jones potentials that were smoothly switched to zero from 1.0 to 1.2 nm. The simulations were conducted at a constant temperature of 323.15 K and pressure of 1 bar using a Nosé–Hoover thermostat [18,19] with a time constant of 1 ps and the Parrinello–Rahman barostat [20], with a period of 5 ps as implemented in GROMACS. The pressure changes were coupled in the x-y dimension, while the z-dimension varied independently. The trajectories evolve following a leapfrog integrator with a time step of 2 fs. Bond lengths to hydrogen atoms in the lipid molecules and peptide were constrained with the LINCS algorithm [21], while the bond distances of the TIP3P water molecules were constrained with the SETTLE algorithm [22].

## 3. Results and Discussions

**Figure 2:**
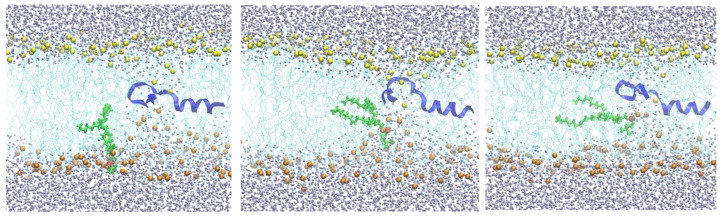
Snapshots of a membrane with NAF-1^44–67^ restrained to the center. The peptide is shown as a blue ribbon, which is partially helical. As in Figure 1, the lipid chains are in cyan. The lower phospholipid heads are in orange, while the top layer is in yellow. The mobile phospholipid is in green while the phosphorus atom is in red. Water molecules are in gray. The three snapshots from left to right illustrate the migration of the phospholipid from the water–membrane interface to the membrane center.

Steepest descent minimization of the initial configurations reduces the maximum force on any atom to less than 1000 kJ/(mol nm). The minimization was followed by a series of equilibration steps for a total of 375 ps in the two systems using the default input files generated by CHARMM-GUI. Equilibration was followed by a longer run for 250 ns. For the system containing NAF-1^44–67^, an additional harmonic restraint with a force constant of 42,000 kJ/(mol nm^2^) was added to the center of mass of NAF-1^44–67^ to keep it at the membrane center. At the end of this simulation with the peptide at the center, the membrane structure was distorted, with about one hundred water molecules entering the membrane core. Several phosphate head groups moved towards the membrane core as well (Figure 2).

**Figure 3:**
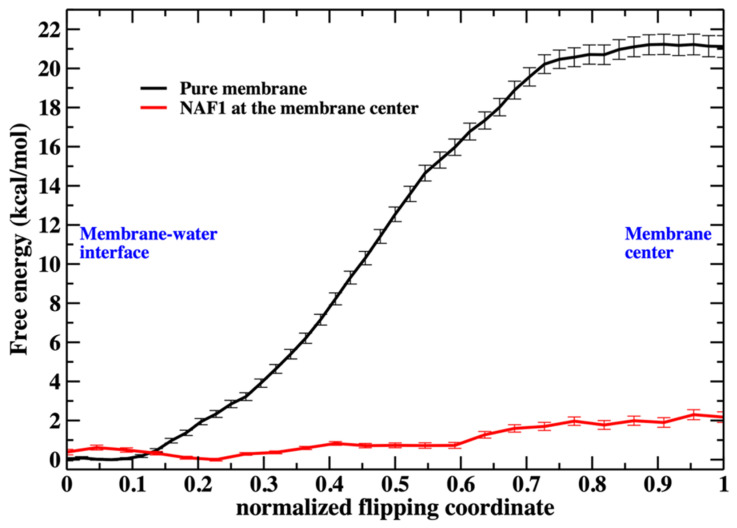
The free energy profile for the migration of a single phospholipid head from the water– membrane interface to the center of the membrane. The red curve is for the membrane containing at the center the NAF-1^44–67^ peptide. The reaction coordinates are parameterized from 0 (phosphate head group at the membrane–water interface) to 1 (the phospholipid head group is at the center of the membrane). See the text for more details about the reaction coordinates.

From the 250 ns trajectory with the peptide inserted (configurations are saved every 100 ps), we tracked one of the phosphate groups from the membrane surface to the center of the membrane (shown in green in Figure 2). The free-energy landscape for phospholipid migration into the lipid domain in the presence of NAF-1^44–67^ is flat (Figure 3), and this choice has little impact on the results. The phosphate-group positions serve as anchors in the Milestoning calculations [23]. The anchors form centers of Voronoi cells in the Milestoning reaction space, and we computed transitions between the interfaces of the Voronoi cells (milestones).

As we illustrate in Figure 3, the barrier height for a phospholipid flip in the system with the peptide is low. We therefore used a simple reaction coordinate, the distance along z (the normal to the membrane plane) of the phosphorus atom from the membrane center. Milestoning employs this coordinate for the system containing NAF-1^44–67^ at the membrane center. The position of the membrane center is computed as the average position along z of the terminal methyl of the phospholipid tails. Twenty-four configurations were selected (Milestoning anchors) from a displacement of 2.2 to −0.1 nm to the membrane center.

Saving intermediate trajectory snapshots of a phospholipid approaching the center of the membrane is straightforward in the simulation that includes the peptide, as the free-energy barrier is low (Figure 3). This is because the peptide itself causes significant membrane disruption. Without the peptide, the displacement of a phospholipid head into the membrane center is an activated process and a rare event with a barrier height of about 20 kcal/mol. A similar barrier height for the same membrane system has been obtained by others [4].

**Figure 4:**
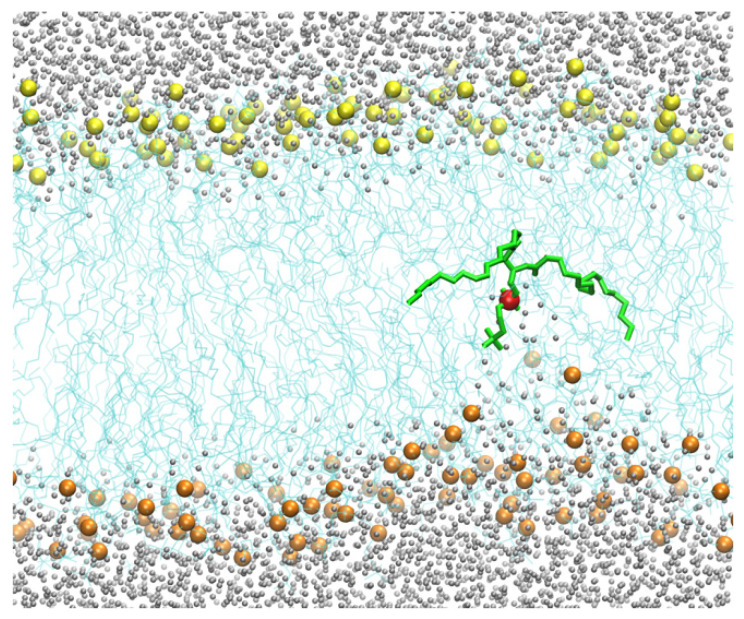
The lower layer of the membrane is distorted, but the upper layer remains unperturbed when a single DOPC molecule is pulled to the center of the membrane from the lower layer. This observation suggests that the system is not in equilibrium, in which both sides of the membrane on either side of the permeating phospholipid should be similarly distorted. Hence, this simulation suffers from hysteresis. The color code is the same as in Figure 2.

Therefore, for the pure DOPC membrane system, we added a harmonic pulling potential that forced the position of one of the phosphorus atoms from the membrane–water interface to the membrane center. The barrier, without the defects created by the CPP, is significantly larger and challenging to converge in free-energy calculations. The pulling creates a membrane distortion that only affects the layer containing the displaced phosphate group (Figure 4). As the phospholipid head approaches the membrane center, the asymmetry of water penetration indicates that the system is not in equilibrium on the time scale of the pulling. The displaced phospholipid “remembers” the layer it originated from, a phenomenon called hysteresis. At equilibrium, the response should be symmetric when the peptide is at the center.

**Figure 5:**
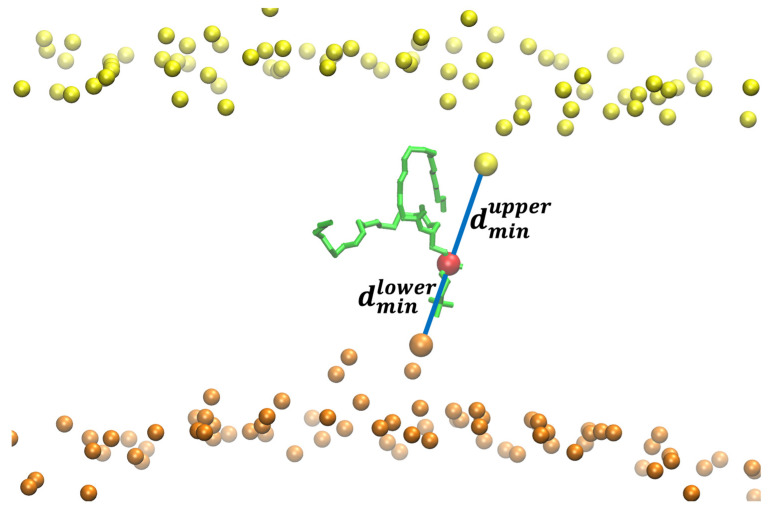
The reaction coordinate used for the flipping of a DOPC phospholipid in a pure DOPC membrane. The figure shows the flipping phospholipid in green with its phosphorus atom in red. The phosphorus atoms are in yellow and orange for the upper and lower layers of the membrane, respectively. The distances between the phosphorus atoms closest to the flipping P atom are shown with blue lines. The difference between $d_{min}^{upper}$ and $d_{min}^{lower}$ is the reaction coordinate ($d_{diff}$) used in the Milestoning calculation. When the difference between the distances is zero, the phospholipid is at the center of the membrane. When the phospholipid is at a water–membrane interface, the absolute value of the difference is at its maximum. For the reaction coordinate, we define the maximal value of the difference as zero (no translocation of the phospholipid to the center) and the minimal distance (displaced phospholipid at the center) as one.

The asymmetry and hysteresis make it hard to converge the exact Milestoning calculations. It produces a non-differentiable free-energy peak at the membrane center. For that reason, we used a different reaction coordinate for the pure phospholipid system (Figure 5). We consider the difference in the minimal distances of the displaced phosphate group to the phosphates in each membrane layer:

(1) $$d_{diff}=d_{min}^{upper}-d_{min}^{lower}$$

where $d_{min}^{upper}$ and $d_{min}^{lower}$ are the minimal distances of the displaced phosphate to the upper- and lower-layer phosphates, respectively. If the head is exactly at the center, the difference is zero, and its absolute value is maximal when one of the distances, upper or lower, is close to zero. We computed the minimum distance with the function

(2) $$d_{min}^{X}=\frac{\beta }{\log\sum_{i}exp\left(\frac{\beta }{r_{i}}\right)}$$

that ensures the minimum distance is differentiable. $r_{i}$ is the distance between the displaced P (phosphorus) atom and every P atom *i* in the layer X, and β is a parameter. A large value of β makes $d_{min}^{X}$ closest to the exact lowest value for the minimal distance. We used β=300 nm in our simulations.

The expectation is that at the transition state $d_{diff}=0$. We enforced this condition with a harmonic restraint and ran a 1 μs trajectory that kept the displaced phosphate close to the membrane center. To verify that the restraint condition created configurations close to the transition state, we performed a committor test. We extracted 20 different configurations from this trajectory and conducted unbiased trajectories. In 12 of those trajectories, the phosphate group drifted towards the lower layer, and in the other eight trajectories, it drifted towards the upper layer, demonstrating that this trajectory was sampling a region close to the transition state for phospholipid flipping (committor ~0.5). Then, we considered one of the 12 trajectories where the phosphate went to the lower layer and extracted 47 anchor configurations for the Milestoning calculation. In the calculation, we used $d_{diff}$ as reaction coordinate with a separation between anchors of about 0.05 nm when $d_{diff}\leq 1.0$ nm, and 0.1 nm for larger $d_{diff}$.

A python script (Python version 3.8) ScMiles2 [23] was used to run and analyze the corresponding Milestoning calculations using GROMACS 2021.4 as the MD engine [14] and PLUMED 2.8 [24] to control and evaluate the positions of the corresponding reaction coordinate during the different Milestoning steps.

In the first Milestoning step (“seek”), we ran short trajectories starting from the set of anchors to determine configurations at connected milestones. For the single Cartesian reaction coordinates considered in this work, milestones are planes equidistant from a neighboring set of anchors. Therefore, from the initial set of 24 anchors used in the Milestoning calculation for the system containing NAF-1^44–67^, configurations were sampled on 23 milestones. For the pure membrane system with 47 anchors, the number of milestones is 46.

In the second Milestoning step (“sample”), we used one of the milestone configurations obtained by the “seek” step and ran a 4 ns restrained trajectory keeping the position of the selected phosphorus atom at the milestone plane. This was accomplished by adding a harmonic potential with a force constant of 42,000 kJ/(mol nm^2^). We saved 500 configurations from these 4 ns trajectories, and used the last 450 configurations as initial configurations for the next Milestoning step (Figure 1 and Figure 2).

In the third step, we ran 450 unrestrained trajectories, starting from the saved configurations, until they were terminated at any other milestone. The Python version 3.8 script saved the identity of the milestone that was hit and the trajectory time. Next, we constructed the transition matrix $W_{ij}$, which provides the transition probability from milestone i to milestone j. We also computed the lifetime of the milestone $t_{i}$, which is the average time it takes the trajectory initiated at milestone i to reach any other milestone. From the matrix $W_{ij}$ we can compute the vector of fluxes between the milestones—$q_{i}$, $q^{t}=q^{t}W$. With the fluxes at hand, we can write the free energy, $F=-kTlog\left(q\cdot t\right)$, and the Mean First Passage Time, $\langle \tau \rangle =\sum_{i}q_{i}\cdot t_{i}/q_{f}$ where $q_{f}$ is the flux at the final absorbing milestone (the product in which the phosphate head group is at the center of the membrane).

**Figure 6:**
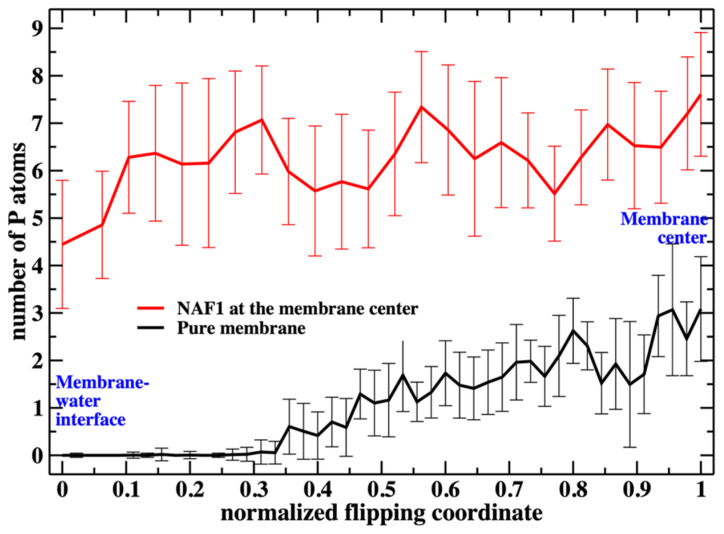
The number of additional phospholipid heads that are at the membrane center as a function of the position of the flipping phospholipid head, which we monitor along the reaction coordinate. Without the peptide at the membrane center, the number is less than 3 when the translocated phospholipid reaches the center. The peptide, with a charge of +5, supports a steady and larger number of phospholipids at the center (between five to seven).

Putting the low barrier in the presence of a CPP in perspective, we note that in the presence of a single CPP in the membrane center, the membrane is already permeable to charged groups such as the phosphate head. Hence, the membrane’s permeability changes once the permeating peptide is inside the center. To further probe the impact of the CPP on membrane structure, we characterize structural features of the defect. In Figure 6, we show the number of phosphate groups along the normalized reaction coordinate.

**Figure 7:**
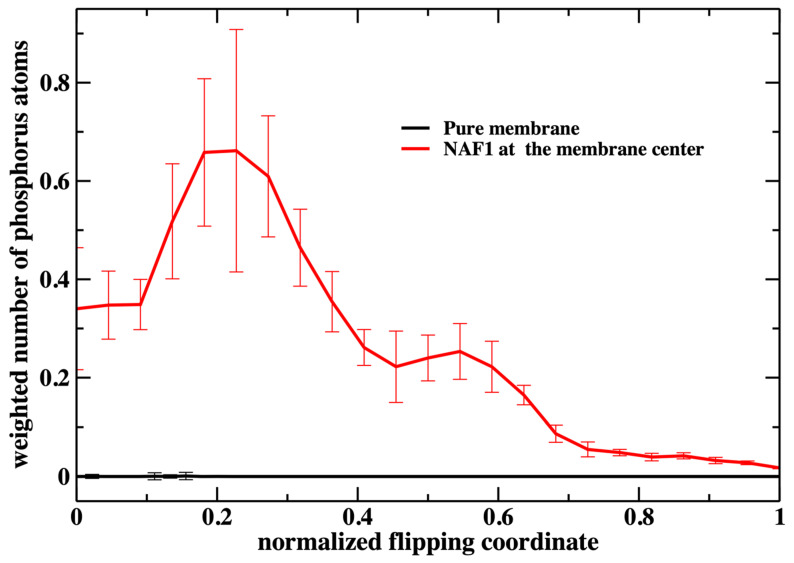
The weighted number of phosphorus atoms at the membrane core along the normalized flipping coordinate. The weight is performed according to the respective milestone probability obtained in our calculations (Figure 3). It is the probability of the translocated phospholipid reaching a position along the reaction coordinate multiplied by the number of phospholipid heads that are at the membrane core (shown in Figure 6). We define the membrane core as the region of the membrane that is less than 1 nm from the membrane center along the z coordinate. This evaluation was performed using the 450 configurations extracted in every milestone (the error bars are obtained as the standard deviation of the average).

Figure 6 shows the number of phospholipids at the membrane center at fixed positions of the phospholipid that follows the reaction coordinate. The plot does not include the work or the probability of bringing the translocated phospholipid to the different positions along the reaction coordinate. This work is shown in Figure 3. In Figure 7, we consider the number of phospholipids at the membrane center multiplied by the probability that the translocated phospholipid will be at a given position along the reaction coordinate (see Figure 3). We call this quantity “weighted number”.

The weighted number of phosphate atoms as a function of the flipping coordinate in the pure DOPC membrane is so small that it is not detectable in Figure 7. This is not surprising given the general feature of the membrane as a barrier. It is more interesting that the presence of a CPP enhances the number of the phosphate atoms slightly shifted into the membrane. The defect created by the CPP is causing the membrane to become somewhat narrower, as is reflected by the peak of the phosphate distribution of a reaction coordinate near 0.2.

**Figure 8:**
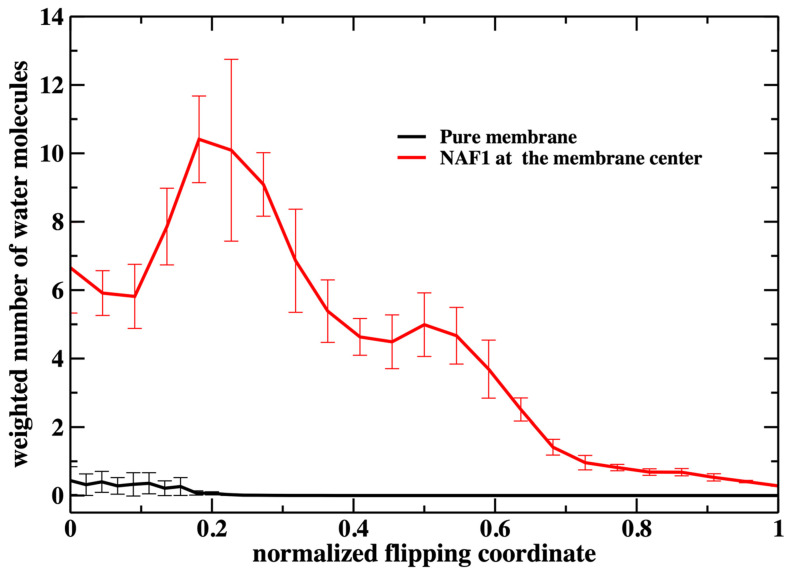
Similar to the previous figure (Figure 7), but counting the number of water molecules in the membrane core for the two membrane systems. A significant difference between the two figures is the permeation of water molecules into the pure membrane. The number of water molecules at the core is about 2 for the unperturbed pure membrane (the weighted number > 0 because the milestone probability is high when the normalized flipping coordinate is close to zero). When the flipping peptide is at the center, the number of water molecules at the core is about 35. However, the milestone probability is very small at that location and the plot looks flat close to one for the pure membrane.

A similar feature of membrane thinning is also seen in Figure 8 in which we examine the number of water molecules along the permeation coordinate. Again, we observe a peak in the number of water molecules inside the membrane at a value of about 0.2. There is some permeation at the water–membrane interface for the pure membrane, which is missing for the phosphate heads in Figure 7. Nevertheless, the number of permeating water molecules is exceptionally small for the pure membrane.

**Figure 9:**
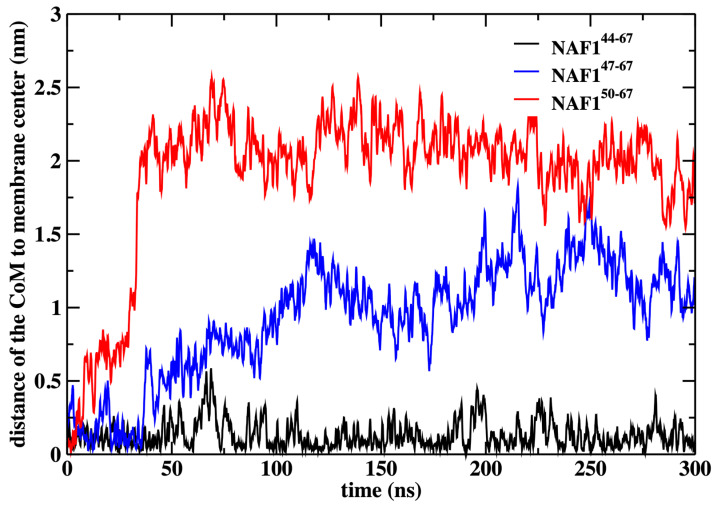
The distance of the center of mass of the peptides NAF-1^44–67^, NAF-1^47–67^, and NAF-1^50–67^ to the membrane center, obtained by 300 ns MD simulations after removing the harmonic restraint that forced the peptides to the membrane center. The simulations illustrate that the original peptide remains in the center while the shorter versions drift away from it.

In this paper, the simulations were conducted with a restraint on the center of mass position of the NAF-1^44–67^ peptide to the membrane center. This restraint improves the statistics of the calculations, but we may ask how probable this configuration is. We have previously computed the potential of mean force for NAF-1^44–67^ permeation across the DOPC membrane, calculations that were supported by experimental data [12]. These calculations have demonstrated that the center of the membrane is a broad minimum for the peptide. To illustrate that the peptide remains in the center even without the restraint, we conducted an independent 300 ns simulation without the restraint starting from the peptide position used in the rest of the paper (Figure 9).

**Figure 10:**
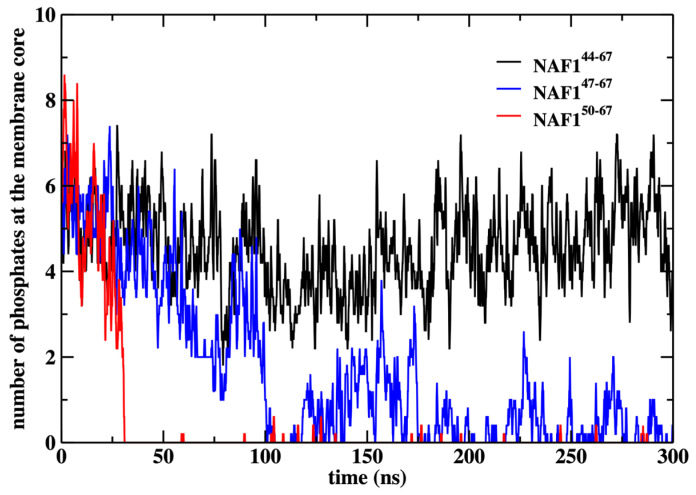
Number of phosphate groups at the membrane core after removing the center of mass restraint and performing a 300 ns MD run for the three peptides NAF-1^44–67^, NAF-1^47–67^, and NAF-1^50–67^. The membrane core is defined as the region within 1 nm of the bilayer center.

We also show that the results are sensitive to the peptide length. Removing three or six residues from the peptide’s N-terminal changes the center of mass location significantly and reduces the number of phospholipids that are dragged to the center (Figure 9 and Figure 10). The last observation supports our initial goal of seeking peptides of minimal length that still assist in creating membrane defects. Hence, NAF-1^44–67^ is close to minimal length for this class of peptides.

In Figure 10, we show the number of phospholipids that are dragged to the membrane center under the influence of the different peptides. Note that the original peptide drags to the center about five phospholipids, which is comparable to the number dragged by the restrained peptide (Figure 6). As the other peptides drift away from the membrane center, their ability to bring phospholipids to the membrane core also reduces.

## 4. Conclusions

In the present manuscript, we examine the process of phospholipid flip-flop, which has long been considered a specialized event in biological systems. Biological membranes are highly asymmetric, and specialized proteins retain this high level of asymmetry, which is assumed to be crucial for membrane function [3].

Because of bilayers’ amphipathic nature, a flip-flop motion is unlikely since it requires the passage of a charged phospholipid across the hydrophobic environment. Indeed, a straightforward estimate of the barrier for a flip-flop suggests a barrier of about 20 kcal/mol. This is sizable but not impossible to cross on the time scale of cell life. Therefore, in biology, additional machinery such as flippases is required [3].

The main topic of this manuscript is, however, flip-flop events that are assisted by membrane perturbations. A significant perturbation creates a defect in which the core is no longer purely hydrophobic, and the barrier for a flip-flop event is reduced to effectively zero. Large perturbations like pore formation are known to accelerate exchanges between the bilayers [6]. However, it is not known how small a perturbation can be, and it still significantly enhances flip-flop rates. We show that an anti-cancer peptide reduces the flip-flop free energy barrier to essentially zero, indicating another plausible function of these intriguing CPP molecules.

More research will have to be carried out to better characterize the required peptide properties and the impact of membrane heterogeneity. However, the current study demonstrates the impact of defects on passive permeation and on flip-flop events that can be classified as permeation events.

## Data Availability

The original contributions presented in this study are included in the article. Further inquiries can be directed to the corresponding author.

## References

[1] Singer S.J., Nicolson G.L. (1972). The Fluid Mosaic Model of the Structure of Cell Membranes. Science.

[2] Bretscher M.S. (1972). Asymmetrical lipid bilayer structure for biological membranes. Nat. New Biol..

[3] Kol M.A., de Kruijff B., de Kroon A. (2002). Phospholipid flip-flop in biogenic membranes: What is needed to connect opposite sides. Semin. Cell Dev. Biol..

[4] Sapay N., Bennett W.F.D., Tieleman D.P. (2009). Thermodynamics of flip-flop and desorption for a systematic series of phosphatidylcholine lipids. Soft Matter.

[5] Clarke R., Hossain K., Cao K. (2020). Physiological roles of transverse lipid asymmetry of animal membranes. Biochim. Biophys. Acta (BBA) Biomembr..

[6] Fattal E., Nir S., Parente R.A., Szoka F.C. (1994). Pore-forming peptides induce rapid phospholipid flip-flop in membranes. Biochemistry.

[7] Doktorova M., Heberle F.A., Marquardt D., Rusinova R., Sanford L., Peyear T., Katsaras J., Feigenson G., Andersen O.S. (2018). Gramicidin Increases Lipid Flip-Flop in Symmetric and Asymmetric Lipid Vesicles. Biophys. J..

[8] Carrer M., Nielsen J.E., Cezar H.M., Lund R., Cascella M., Soares T.A. (2023). Accelerating Lipid Flip-Flop at Low Concentrations: A General Mechanism for Membrane Binding Peptides. J. Phys. Chem. Lett..

[9] Povilaitis S.C., Fathizadeh A., Kogan M., Elber R., Webb L.J. (2022). Design of Peptides for Membrane Insertion: The Critical Role of Charge Separation. J. Phys. Chem. B.

[10] Elber R. (2023). Defect Formation and Peptide Permeation across Phospholipid Membranes. J. Phys. Chem. B.

[11] Park P., Matsubara D.K., Barzotto D.R., Lima F.S., Chaimovich H., Marrink S.J., Cuccovia I.M. (2024). Vesicle protrusion induced by antimicrobial peptides suggests common carpet mechanism for short antimicrobial peptides. Sci. Rep..

[12] Cardenas A.E., Drexler C.I., Nechushtai R., Mittler R., Friedler A., Webb L.J., Elber R. (2022). Peptide Permeation across a Phosphocholine Membrane: An Atomically Detailed Mechanism Determined through Simulations and Supported by Experimentation. J. Phys. Chem. B.

[13] Jo S., Kim T., Im W., Yuan A. (2007). Automated Builder and Database of Protein/Membrane Complexes for Molecular Dynamics Simulations. PLoS ONE.

[14] Abraham M.J., Murtola T., Schulz R., Páll S., Smith J.C., Hess B., Lindahl E. (2015). GROMACS: High Performance Molecular Simulations Through Multi-Level Parallelism From Laptops to Supercomputers. SoftwareX.

[15] Huang J., Rauscher S., Nawrocki G., Ran T., Feig M., de Groot B.L., Grubmüller H., MacKerell A.D. (2017). CHARMM36m: An improved force field for folded and intrinsically disordered proteins. Nat. Methods.

[16] Jorgensen W.L., Chandrasekhar J., Madura J.D., Impey R.W., Klein M.L. (1983). Comparison of simple potential functions for simulating liquid water. J. Chem. Phys..

[17] Essmann U., Perera L., Berkowitz M.L., Darden T., Lee H., Pedersen L.G. (1995). A smooth particle mesh Ewald method. J. Chem. Phys..

[18] Hoover W.G. (1985). Canonical Dynamics—Equilibrium Phase-Space Distributions. Phys. Rev. A.

[19] Nose S. (1984). A Molecular-Dynamics Method for Simulations in the Canonical Ensemble. Mol. Phys..

[20] Parrinello M., Rahman A. (1981). Polymorphic Transitions in Single-Crystals—A New Molecular-Dynamics Method. J. Appl. Phys..

[21] Hess B., Bekker H., Berendsen H.J., Fraaije J.G. (1997). LINCS: A linear constraint solver for molecular simulations. J. Comput. Chem..

[22] Miyamoto S., Kollman P.A. (1992). Settle—An Analytical Version of the Shake and Rattle Algorithm for Rigid Water Models. J. Comput. Chem..

[23] Cardenas A.E., Hunter A., Wang H., Elber R. (2022). ScMiles2: A Script to Conduct and Analyze Milestoning Trajectories for Long Time Dynamics. J. Chem. Theory Comput..

[24] Tribello G.A., Bonomi M., Branduardi D., Camilloni C., Bussi G. (2014). PLUMED 2: New feathers for an old bird. Comput. Phys. Commun..

